# Association of nutrient intake and dietary patterns with serum folate and anemia-related biomarkers in Taiwanese pregnant women with pre-pregnancy overweightness or obesity

**DOI:** 10.7150/ijms.108760

**Published:** 2025-02-28

**Authors:** Nhi Thi Hong Nguyen, Chyi-Huey Bai, Jung-Su Chang, Yi-Chun Chen, Ya-Li Huang, Fan-Fen Wang, Chien-Yeh Hsu, Arpita Das, Jane C.-J. Chao

**Affiliations:** 1 School of Nutrition and Health Sciences, Taipei Medical University, Taipei, Taiwan.; 2 Health Personnel Training Institute, University of Medicine and Pharmacy, Hue University, Hue city, Vietnam.; 3 Department of Public Health, School of Medicine, Taipei Medical University, Taipei, Taiwan.; 4 School of Public Health, Taipei Medical University, Taipei, Taiwan.; 5 Nutrition Research Center, Taipei Medical University Hospital, Taipei, Taiwan.; 6 Graduate Institute of Metabolism and Obesity Sciences, Taipei Medical University, Taipei, Taiwan.; 7 TMU Research Center for Digestive Medicine, Taipei Medical University, Taipei, Taiwan.; 8 Department of Internal Medicine, Yangming Branch, Taipei City Hospital, Taipei, Taiwan.; 9 Department of Information Management, National Taipei University of Nursing and Health Sciences, Taipei, Taiwan.; 10 Master Program in Global Health and Health Security, Taipei Medical University, Taipei, Taiwan.

**Keywords:** pregnant women, serum folate, iron biomarkers, dietary pattern, overweight, obesity

## Abstract

Pre-pregnancy overweightness or obesity affects nutritional status and micronutrient imbalance such as folate and iron during pregnancy. We studied the relationships between micronutrient intake and dietary patterns with serum folate and iron biomarkers among pregnant population with pre-pregnancy overweightness or obesity. This cross-sectional study utilized data from 2017-2019 Nationwide Nutrition and Health Survey. Participants (*n* = 436) were Taiwanese pregnant women with pre-pregnancy overweightness or obesity. Dietary evaluation was conducted by food frequency questionnaire and 24-hour dietary recall. Dietary patterns were developed by principal component analysis. Serum folate and iron biomarkers were measured. Logistic and linear regression models were employed to investigate the associations of dietary patterns with serum folate and iron biomarkers. Participants with the highest tertile of serum folate were older, and had less proportion of high parity. After adjusting covariates, the intake of DP-1 (mushrooms, roots, and dairy DP) (β = 0.052; 95% CI = 0.008-0.119; *p* < 0.05) or DP-3 (vegetables and fruits DP) (β = 0.056; 95% CI = 0.034-0.076; *p* < 0.05) was associated with increased serum folate. The consumption of DP-4 (animal DP) showed a significant association with elevated serum iron, ferritin, and vitamin B-12. The consumption of DP-1 or DP-3 was linked to a decreased risk of low serum folate. However, the intake of DP-3 was correlated with an increased risk of low serum ferritin. A reduced risk of low serum iron and vitamin B-12 was observed in participants consuming DP-4. The dairy and plant dietary patterns were positively associated with serum folate, and the animal dietary pattern was positively correlated with serum iron and vitamin B-12 in pregnant women with pre-pregnancy overweightness or obesity.

## Introduction

Both maternal and fetal health could be influenced by the nutritional status during the periods of pre-pregnancy and pregnancy in expected mothers 1. Folate deficiency during pregnancy could increase the risk of neural tube defects in the fetus. An additional folate requirement (200 µg) above daily regular requirement of 400 µg for pregnant women was recommended by the Ministry of Health and Welfare (Taiwan) 2. Women with overweightness or obesity were correlated to reduced serum folate levels 3, and obese women in pre-pregnancy used less folic acid supplement before pregnancy than their counterparts with normal body mass index (BMI) 4. Additionally, overweight or obese women in pre-pregnancy were associated with increased odds of having preterm delivery compared with those with normal BMI, and with a reduced risk of preterm delivery if they had folic acid supplement from the 1st trimester of the pregnancy 5.

Maternal vitamin B_12_, vitamin D, and iron status during pregnancy is important for fetal growth and development. Obese women in pre-pregnancy had lower serum vitamin B-12 levels during pregnancy compared with those with normal BMI 6. Vitamin D deficiency in pregnant women was concerned for public health issue, and crucial to prevent maternal vitamin D deficiency for better infant outcomes 7. Obesity could be correlated to low serum vitamin D status 8. Additionally, maternal obesity during pregnancy was reported to raise the risk of having insufficient vitamin D levels for both mothers and infants 9, and vitamin D deficiency was associated with an increased risk of preeclampsia 10. Recently, maternal obesity has been indicated to have an association with heightened risk of iron deficiency during pregnancy and iron-deficiency anemia in both mothers and infants 11.

The previous studies investigating the dietary habits of pregnant women with overweightness or obesity have primarily concentrated on dietary patterns, energy consumption, and macronutrient intake 12-14. Furthermore, the research exploring micronutrient intake in pregnant women has focused on the undernourished pregnant women instead of the pregnant women with overweightness or obesity 15. The study for the utilization of dietary pattern (DP) analysis to comprehend the risk factors related to micronutrient deficiencies during pregnancy remains restricted. Therefore, our study aimed to identify the associated factors of micronutrient intake and DPs with serum folate levels and iron biomarkers in pregnant women with pre-pregnancy overweightness or obesity.

## Material and Methods

### Data source

The subjects in the Nationwide Nutrition and Health Survey were pregnant individuals and recruited from 11 hospitals using stratified sampling in Taiwan from 2017 to 2019 16.

### Study population

The inclusion criteria for recruited pregnant women were those who had (1) age of 15 years or above, (2) the maternal health assessment booklet, (3) obstetric examination services more than once, (4) the ability to communicate in Mandarin or Taiwanese, (5) the willing to join the study and sign the consent form (for those who were under 20 years, parental permission was obtained from the informed agreement), and (5) pre-pregnancy BMI ≥ 24 kg/m^2^. Pre-pregnancy BMI was computed by dividing body weight (kg) by height (m^2^), and classified as overweight or obesity if the BMI value was ≥ 24 kg/m² 17. Participants having non-singleton pregnancies, multiparity (> 4), or being nonresponsive were excluded from the study. A total of 436 participants were finally incorporated in the analysis.

### Dietary assessment

The dietary assessment was performed by a standardized semi-quantitative food frequency questionnaire (FFQ) derived from the NAHSIT FFQ 18, and by a 24-hour dietary recall method. The supplement intake was also recorded as monthly frequency using the questionnaire. The FFQ was utilized to identify the habitual dietary consumption of a total of 59 food items as described in detail elsewhere 18. The consumption frequency of each food item in the FFQ was evaluated according to daily, weekly, or monthly consumption, and the overall monthly frequency of a specific food category was finally calculated. To calculate the dietary pattern (DP) score, 59-item in the FFQ was further derived into 26 food groups 18. The food items with comparable nutrients were assigned to the same food group (Supplementary Table S1). Different DPs were determined by principal component analysis (PCA), a multivariate technique for analyzing variation and categorizing strong patterns in the dataset 19. The 24-hour dietary recall data were assessed by the well-trained registered dietitian via face-to-face interview. Nutrient intake was standardized by Taiwan's food nutrient database, and calculated using Cofit Pro (Cofit Healthcare, Taipei, Taiwan).

### Assessment of anemia-related biomarkers

After blood samples were collected at the baseline during the prenatal visit, serum biomarkers associated with both micronutrient intake and iron biomarkers including folate, vitamin B_12_, 25(OH) vitamin D, hemoglobin (Hb), iron, ferritin, total iron-binding capacity (TIBC), and transferrin saturation were assessed.

Serum folate 20 and vitamin B_12_ concentrations 21 were evaluated by a SimulTRAC-SNB radioimmunoassay kit (MB Biomedicals, Santa Ana, CA) with ^125^I or ^57^Co as the tracer. Serum levels of 25(OH) vitamin D were assessed utilizing the Elecsys vitamin D total reagent kit by an electrochemiluminescence immunoassay with ruthenium-labeled vitamin-D-binding protein (Roche Diagnostics Ltd., Taipei, Taiwan) 22. Serum Hb levels (g/dL) were measured using a hematology analyzer from Sysmex Corp., Kobe, Japan. Serum iron concentrations (µmol/L) were analyzed by a ferrozine-based colorimetric assay. Serum ferritin was evaluated by a chemiluminescence immunoassay. Additionally, TIBC levels (µmol/L) were identified by an immunoturbidimetric method. These indicators (serum iron, ferritin, and TIBC) were assessed using a Beckman Coulter Unicel DxC 800 instrument 23. Transferrin saturation (%) was calculated by dividing serum iron levels by TIBC value and multiplying by 100% 24.

We categorized these gestational anemia-related biomarkers into either normal or abnormal levels using the clinical cutoffs established by the World Health Organization and the Centers for Disease Control and Prevention. For serum folate across all age groups, the standard reference range was 13.6-45.3 nmol/L (6-20 ng/mL) 25. The cutoff for vitamin B_12_ deficiency was defined as < 149.8 pmol/L (< 203 pg/mL) 26. Serum 25(OH) vitamin D was classified as a deficiency if its concentration was < 75 nmol/L (< 30 ng/mL) 27. The standard reference ranges for serum iron and TIBC in non-anemic women were 10.7 µmol/L (60 µg/dL) 28 and 42.96-80.55 µmol/L (240-450 µg/dL), respectively 29. Gestational anemia was identified as serum ferritin level <0.034 nmol/L (< 15 µg/L) 30, and transferrin saturation < 16% 31.

### Assessment of covariates

Information about sociodemographic data and daily micronutrient intake was assessed at the baseline. Sociodemographic factors including age (years), region of residence (northern, central, southern, eastern, and other), education level (high school, undergraduate school, or graduate school), parity (1, 2, or ≥ 3), number of pregnancies, household income (NTD), and gestational trimester (first: 0-12 weeks, second: 13-26 weeks, and third: 27-40 weeks) 32. Daily micronutrient intake comprised the consumption of energy (kcal), macronutrients (g and % of energy), folate (µg), vitamin B_12_ (µg), vitamin D (µg), and iron (mg).

### Statistical analysis

Participants' baseline characteristics were compared across serum folate tertiles using Krusal-Wallis test for continuous variables and chi-square test for categorical variables. Kolmogorov-Smirnov test was applied to determine the distribution of the data. Spearman correlation coefficients were computed between micronutrient intake and serum folate levels. The PCA method was performed by PROC PLS tool in SAS 9.4 (SAS, Cary, NC, USA) to categorize 26 food groups into 4 DPs. Factor loadings less than 0.30 were eliminated to streamline the analysis 33. High factor loadings highlight robust associations of food groups with disease.

We identified the associations of the DPs with iron biomarker levels by generalized linear regression analysis for computing β values and 95% confidence intervals (CIs). Model 1 was a crude model. Model 2 was adjusted for sociodemographic factors. Model 3 was adjusted for model 2 and daily nutrient consumption. Next, we classified the DP scores in each DP into 3 equally dispersed groups tertiles 1-3 (T1-T3) with increased DP scores, and T1 was designated as the reference group.

Subsequently, we determined the odds ratio (OR) and its respective 95% CIs to evaluate the relationship between DPs (across the tertiles of each DP) and abnormal levels of serum biomarkers. This analysis employed the multivariable logistic regression with adjustments for the identical covariates incorporated in the generalized linear regression models mentioned previously. Incorporating categorical variables into the model involved dummy coding where binary variables were generated to represent every category within the categorical variables. The reference group for region of residence, education level, parity, or trimester was northern region, high school, 1 parity, or trimester first, respectively. Analyses were performed utilizing R programming software (version 4.1.3, R Development Core Team, Vienna, Austria). All *p* values of ≤ 0.05 were identified as the statistical significance.

## Results

### Subject characteristics

Table 1 presents participants' characteristics stratified by the tertiles of serum folate levels. Individuals in T3 of serum folate had the highest average age. The participants in T3 of serum folate had a higher proportion of being resident in the northern region (35.6%), having an undergraduate degree (74.5%), having one parity (52.1%), and being in trimester first (31.5%) compared to those in other tertiles.

The participants in T3 of serum folate (46.7 ± 7.2 nmol/L) tended to have elevated levels of serum vitamin B_12_ (325.3 ± 139.0 pmol/L), iron (14.9 ± 7.0 mmol/L), and ferritin (0.07 ± 0.06 nmol/L), however, their TIBC levels were comparatively lower (78.9 ± 16.2 µmol/L) (Table 2).

### Association of nutrient intake with serum folate levels

Table 3 indicates Spearman's correlation coefficients of daily nutrient intake with serum folate levels among pregnant women with pre-pregnancy overweightness or obesity. The intakes of protein in terms of g (*ρ*: 0.18; *p* = 0.044) or % of energy (*ρ*: 0.15; *p* = 0.002), folate (*ρ*: 0.12; *p* = 0.010), and iron (*ρ*: 0.11; *p* = 0.018) were positively correlated to serum folate levels. Supplement intake including multivitamins/multiminerals (*ρ*: 0.39;* p* < 0.001), folate (*ρ*: 0.50; *p* = 0.042), and calcium (*ρ*: 0.12; *p* = 0.043) had a positive correlation with serum folate levels (Supplementary Table 2). Milk powder, vitamin B complex, vitamin D, and iron supplements were not evaluated because the consumption frequency was below 20%.

### Dietary patterns

**Figure 1:**
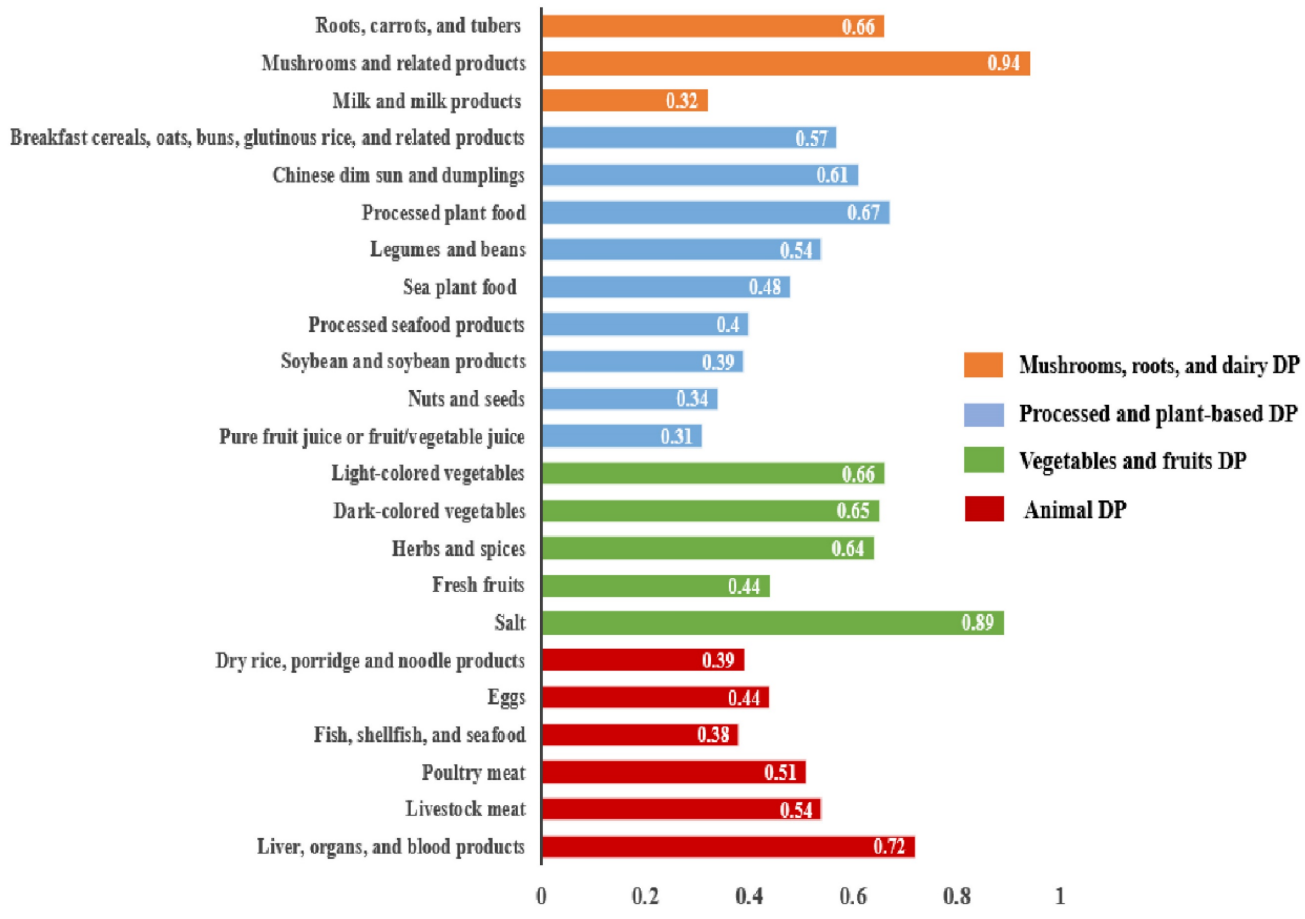
Four distinct dietary patterns derived from principal component analysis.

The PCA results indicated 4 distinct DPs shown in Figure 1. These 4 DPs accounted for a total variance of 10.1% (2.8%, 2.6%, 2.5%, and 2.2%, respectively). DPs were classified and ranked based on a threshold factor loading value of > 0.30. Each DP was named regarding its respective factor loading values and the underlying structure of dietary components. The DP-1 was named as mushrooms, roots, and dairy DP, consisting of 3 food groups: mushrooms and related products; roots, carrots, and tubers; and milk and milk products. The DP-2 was named as processed and plant-based DP. It encompassed 9 food groups: processed plant foods; Chinese dim sun and dumplings; breakfast, cereals, oats, buns, glutinous rice, and related products; legumes and beans; sea plant food; processed seafood products; soybean and soybean products; nuts and seeds; and pure fruit juice or fruit/vegetable juice. The DP-3 was named as vegetables and fruits DP, comprising 5 food groups: salt; light-colored vegetables; dark-colored vegetables; herbs and spices; and fresh fruits. The DP-4 was named as animal DP which consisted of 6 food groups: liver, organs, and blood products; livestock meat; poultry meat; eggs; dry rice, porridge, and noodle products; and fish, shellfish, and seafood.

### Associations of DPs with serum anemia-related biomarkers

Table 4 shows the association of mushrooms, roots, and dairy DP (DP-1) with serum anemia-related biomarkers among pregnant women with pre-pregnancy overweightness or obesity. In all 3 models, the DP-1 was positively associated with an elevation in serum folate levels, with values ranging from 0.038 to 0.052 nmol/L across the 3 models. Additionally, the DP-1 was linked to a 0.004 nmol/L reduction in serum ferritin level in model 1 (β = -0.004, 95% CI = -0.008-(-0.001); *p* < 0.05), however, there was no association after covariate adjustment. The processed and plant-based DP (DP-2) presented no association with anemia-related biomarkers (Supplementary Table 3).

The DP rich in vegetables and fruits** (**DP-3) exhibited increased associations in serum folate levels by 0.039 nmol/L, 0.049 nmol/L, and 0.056 nmol/L in models 1-3, respectively (Table 5). The DP-3 was related to a decrease in serum iron levels by 0.031 µmol/L in model 1 (95% CI = -0.052- (-0.010); *p* < 0.05), and a decline in serum ferritin levels ranging between 0.015 and 0.016 nmol/L in all 3 models.

Animal DP (DP-4) exhibited the associations with anemia-related biomarkers among pregnant women with pre-pregnancy overweightness or obesity (Table 6). Across the 3 models, the DP-4 was associated with elevated serum vitamin B_12_ levels by 0.244-0.321 pmol/L. Correspondingly, the DP-4 was also correlated to increased serum iron levels by 0.015-0.025 µmol/L and elevated serum ferritin levels by 0.014-0.022 µmol/L across the 3 models.

### Associations of DPs with the risk of low serum anemia-related biomarkers

The results of the binomial logistic regression analysis for the relationship between DP-1 and the risk of low anemia-related biomarkers among pregnant women with pre-pregnancy overweightness or obesity are presented in Table 7. The participants consuming DP-1 at higher levels (T2 and/or T3) demonstrated a lower risk of low serum folate levels than those who had less DP-1 consumption (T1) in all 3 models. In model 1, the participants with a higher consumption level (T2) of DP-1 showed a reduced risk of low serum ferritin levels (OR = 0.806, 95% CI = 0.604-0.976; *p* < 0.05) compared to those consumed lower DP-1 (T1), however, no significant associations were observed after adjusting for covariates. The DP-2 did not indicate foremost relationships with serum anemia-related biochemical indicators, except for serum folate levels (Supplementary Table 4). The respondents consuming higher levels (T2 and/or T3) of DP-2 had a decreased risk of low serum folate levels in all 3 models.

Table 8 depicts the association of DP-3 with the risk of low anemia-related biomarkers among pregnant women with pre-pregnancy overweightness or obesity. In all 3 models, the participants with increased DP-3 intake (T2 and T3) were associated with a decreased risk of low serum folate. The subjects with the highest consumption (T3) of DP-3 were correlated to an increased risk of low serum vitamin B_12_ (OR = 2.115, 95% CI = 1.036-4.321; *p <* 0.05) in model 1. The participants with the highest intake (T3) of DP-3 were linked to an elevated risk of low serum ferritin levels with ORs of 2.390 (95% CI = 1.491-3.833; *p <* 0.05) in model 1, 2.414 (95% CI = 1.391-4.189; *p <* 0.05) in model 2, and 2.367 (95% CI = 1.356-4.132; *p <* 0.05) in model 3.

The relationship between DP-4 and the risk of low anemia-related biomarkers among pregnant women with pre-pregnancy overweightness or obesity is displayed in Table 9. The participants with higher intake (T2 and T3) of DP-4 were correlated to a decreased likelihood of low serum vitamin B_12_ levels in both models 2 and 3, and associated with a reduced risk of low serum iron levels in all 3 models.

## Discussion

Our study highlighted significant associations of nutrient intake such as protein, folate, and iron with serum folate levels in pregnant women with pre-pregnancy overweightness or obesity. Additionally, serum vitamin B_12_, iron, ferritin, and TIBC exhibited positive associations with serum folate. These findings are supported by previous studies. Iron-deficiency anemia was identified as being related to low serum folate levels in pregnant women 34, and the normalization of folate distribution between plasma and erythrocytes was observed with oral iron treatment only 35, suggesting that iron intake could be positively correlated with serum folate status. Furthermore, a substantial prevalence of serum folate insufficiency was found in iron-deficiency anemia patients, and could be improved with intravenous iron treatment 36. The interaction of iron and folate absorption may be promoted by heme carrier protein 1 that acts as a proton-coupled folate transporter 37. Our finding showed that serum vitamin D status tended to be increased with elevated serum folate levels, but did not reach to the significant level (*P* = 0.051). A previous study found that serum vitamin D levels were not correlated to serum folate levels (*r* = 0.09) in pregnant women 38.

Our study indicated that the intake of DP-1 (mushrooms, roots, and dairy DP) and DP-3 (vegetables and fruits DP) were associated with an increase in serum folate levels. These findings are supported by previous studies. Vegetables, fruits, legumes, and cereals are recognized as the primary dietary sources of folate 39. Pregnant women who had knowledge about folate-rich foods were observed to be less likely to experience folate deficiency 40. A DP rich in vegetables and fruits positively influenced folate intake during pregnancy 41, 42. Additionally, dietary folate intake was found to be associated with serum folate levels in pregnant women 43. Pregnant women with obesity were found to be associated with insufficient DP of vegetables 44. Women with pre-pregnancy overweightness and obesity were reported to have lower serum folate and an increased risk of serum folate deficiency 45. Pregnant women with ovo-lacto vegetarian had higher plasma folate concentration, and a lower risk of folate deficiency 46. Since serum folate concentration could reflect recent dietary folate intake 47, the associations we identified between the DP and serum folate levels are likely to be comparable and reasonable.

The current study observed an association between the consumption of fruits and vegetables and a decrease in serum ferritin, but no such association was found with serum hemoglobin levels. Flynn *et al.* (2018) observed no difference in serum ferritin levels between the pregnant women with obesity and those with normal weight 48. Serum ferritin serves as an indicator of iron status during pregnancy 49. Fruits and vegetables, rich in dietary fiber, are good sources of nonheme iron which could be absorbed less efficiently in the intestine compared to heme iron 50 due to the inhibitory effect of phytates co-existing in fruits and vegetables on nonheme iron absorption 51. In addition, polyphenols presented in fruits and vegetables could also decrease iron absorption and further lower ferritin concentrations 52. Our study revealed that the consumption of DP-4 exhibited a significant reduction in the risk of low serum iron and vitamin B_12_. Animal foods are the most abundant sources of bioavailable dietary iron. Approximately 40% of total iron in meat, fish, and poultry is in the heme form, which is more readily absorbed than non-heme iron 53. The consumption of iron-rich food from fish and seafood was commonly reported, while the intake of other animal sources of iron, such as organ meat and flesh meat, was less among pregnant women from a rural area in Northern Ghana 54. Higher red and processed meat consumption had a positive association with increased levels of proinflammatory markers among women with overweightness and obesity 55. Adiposity-driven low-grade inflammation reduced iron bioavailability by elevating hepcidin levels, which inhibited iron absorption and efflux 56, 57. Low intake of iron-rich foods from animal sources could potentially increase the risk of iron-deficiency anemia among pregnant women 54. Women with pre-pregnancy overweightness or obesity had increased risks of developing iron and vitamin B_12_ deficiencies compared to those with normal weight 45, 58. In addition, pregnant women adhering to carnivore DP exhibited a reduced risk of low serum vitamin B_12_ 59. A low-animal-product diet increased the risk of vitamin B_12_ insufficiency because nutrient-dense animal foods are major sources of vitamin B_12_ 60. A previous study also indicated that higher intakes of meat and fish were positively associated with increased plasma concentration of vitamin B_12_ and a reduced risk of vitamin B_12_ deficiency in pregnant Dutch women 61.

The current study has several strengths. First, our study undertook a comprehensive assessment of both micronutrient intake and DPs with serum folate status and iron biomarkers in a well-defined population. The novel evidence of our results could assist healthcare professionals and pregnant women in making informed decisions regarding the consumption of dietary supplement. Second, data were collected from diverse regions in Taiwan, and served as a representative sample for the Taiwanese population.

Nonetheless, our study comes with certain limitations. First, we depended on self-administrated dietary data such as FFQ, which could introduce systematic under-reporting or recall bias in energy and/or nutrient consumption. To address and mitigate the biases associated with the FFQ, we took additional measures by collecting 24-hour dietary recall data. Second, certain confounders such as pathological conditions, parathyroid hormone levels, hepcidin levels, and intake of vitamin C among these pregnant women were not available for our analysis. Third, this study could not incorporate some lifestyle factors such as physical activity, cigarette smoking, and alcohol use.

## Conclusions

The intake of protein, folate, and iron was associated with serum folate levels. The consumption of DP-1 (mushrooms, roots, and dairy dietary pattern) or DP-3 (vegetables and fruits dietary pattern) was associated with an elevation in serum folate levels. Additionally, the intake of DP-4 (animal dietary pattern) exhibited a significant association with higher levels of serum iron, ferritin, and vitamin B_12_.

DP-1 consumption was associated with a decreased risk of low serum folate. DP-3 use had an association with an increased risk of low serum ferritin, but with a reduced odds of low serum folate levels. Pregnant women consuming DP-4 had a lower risk of low serum iron and vitamin B_12_ levels. Our findings could contribute to raising awareness on this topic for healthcare professionals and researchers, and provide novel evidence for further investigations to confirm the associations of DPs with iron biomarkers among pregnant women with pre-pregnancy overweightness or obesity.

## Supplementary Material

Supplementary tables.

## Figures and Tables

**Table 1:** Characteristics of pregnant women with pre-pregnancy overweightness or obesity across the tertiles of serum folate (*n* = 436)

Variables	Total (*n*)	Tertiles of serum folate			*p*	
		T1 (*n* = 140- 145)	T2 (*n* = 140- 146)	T3 (*n* = 141-146)		
Age, years	436	31.9 ± 5.2	32.9 ± 4.9	33.9 ± 4.9		0.003
Region of residence, *n* (%)	435					< 0.001
Northern	116 (26.7)	26 (8.1)	38 (26.2)	52 (35.6)		
Central	108 (24.8)	45 (31.2)	36 (24.8)	27 (18.5)		
Southern	98 (22.5)	22 (15.3)	33 (23.1)	43 (29.5)		
Easter and other	113 (26.0)	51 (35.4)	38 (25.9)	24 (16.4)		
Education level, *n* (%)	435					< 0.001
High school	90 (20.7)	50 (34.5)	24 (16.7)	16 (11.0)		
Undergraduate	297 (68.3)	86 (59.3)	104 (70.1)	107 (74.5)		
Graduate	48 (11.0)	9 (6.2)	18 (13.2)	21 (14.5)		
Parity, *n* (%)	434					< 0.001
1	195 (44.9)	45 (31.0)	75 (51.7)	75 (52.1)		
2	183 (42.2)	68 (46.9)	57 (39.3)	58 (40.3)		
≥ 3	56 (12.9)	32 (22.1)	13 (9.0)	11 (7.6)		
Number of pregnancies, *n* (%)	424					0.699
1	413 (97.4)	140 (97.9)	137 (97.9)	136 (96.5)		
≥ 2	11 (2.6)	3 (2.1)	3 (2.1)	5 (3.5)		
Household income, *n* (%)	424					0.160
< 30,000 NTD	63 (14.8)	28 (20.0)	13 (9.2)	22 (15.4)		
30,000-60,000 NTD	197 (46.5)	61 (43.6)	71 (50.4)	65 (45.5)		
≥ 60,000 NTD	164 (38.7)	51 (36.4)	57 (40.4)	56 (39.1)		
Trimesters, *n* (%)	436					0.003
First	104 (23.8)	25 (17.2)	33 (22.8)	46 (31.5)		
Second	146 (33.5)	41 (28.3)	56 (38.6)	49 (33.6)		
Third	186 (42.7)	79 (54.5)	56 (38.6)	51 (34.9)		
						

Data are given as mean ± SD or *n* (%). Kruskal-Wallis test was used for continuous variables, and chi-square test was used for categorical variables. SD: standard deviation; T: tertile.

**Table 2:** Biochemical characteristics of pregnant women with pre-pregnancy overweightness or obesity across the tertiles of serum folate (*n* = 436)

Serum variables	Tertiles of serum folate			
	T1 (*n* = 145)	T2 (*n* = 145)	T3 (*n* = 146)	*p*
Folate, nmol/L	10.9 ± 3.8	24.8 ± 4.2	46.7 ± 7.2	<0.05
Vitamin B_12_, pmol/L	240.2 ± 129.8	298.1 ± 146.9	325.3 ± 139.0	<0.05
25(OH) Vitamin D, nmol/L	60.6 ± 20.2	65.1 ± 19.2	70.8 ± 23.1	0.051
Hemoglobin, mmol/L	7.2 ± 1.3	7.5 ± 1.3	7.5 ± 1.2	0.070
Iron, µmol/L	9.6 ± 4.5	11.9 ± 4.9	14.9 ± 7.0	<0.05
Ferritin, nmol/L	0.04 ± 0.06	0.05 ± 0.05	0.07 ± 0.06	<0.05
TIBC, µmol/L	90.6 ± 19.8	82.3 ± 15.6	78.9 ± 16.2	<0.05
Transferrin saturation, %	18.0 ± 10.7	16.1 ± 9.5	16.3 ± 8.9	0.172

Data are given as mean ± SD. Kruskal-Wallis test was used for continuous variables. SD: standard deviation; T: tertile; TIBC: total iron-binding capacity

**Table 3:** Spearman's correlation coefficients between nutrient intake and serum folate levels among pregnant women with pre-pregnancy overweightness or obesity (*n* = 436)

Nutrient intake	Serum folate	
	*ρ*	*p*
Energy, kcal	0.01	0.785
Carbohydrate, g	-0.03	0.943
Carbohydrate, % of energy	-0.05	0.921
Protein, g	0.18	0.044
Protein, % of energy	0.15	0.002
Fat, g	-0.02	0.615
Fat, % of energy	-0.04	0.461
Folate, µg	0.12	0.010
Vitamin B_12_, µg	0.03	0.499
Vitamin D, µg	0.02	0.959
Iron, mg	0.11	0.018

**Table 4:** Generalized linear regression analysis for the association of mushrooms, roots, and dairy DP (DP-1) with serum anemia-related biomarkers among pregnant women with pre-pregnancy overweightness or obesity (*n* = 436)

Variables	Model 1	Model 2	Model 3
	β (95% CI)	β (95% CI)	β (95% CI)
Folate, nmol/L	0.038 (0.007, 0.112)*	0.052 (0.008, 0.118)*	0.052 (0.008, 0.119)*
Vitamin B_12_, pmol/L	-0.536 (-1.160, 0.089)	-0.328 (-0.921, 0.265)	-0.287 (-0.888, 0.314)
25(OH) Vitamin D, nmol/L	0.027 (-0.011, 0.064)	0.022 (-0.002, 0.006)	0.024 (-0.068, 0.117)
Hemoglobin, mmol/L	-0.003 (-0.008, 0.003)	-0.001 (-0.006, 0.005)	0.001 (-0.006, 0.005)
Iron, µmol/L	-0.007 (-0.034, 0.019)	-0.001 (-0.024, 0.027)	-0.002 (-0.003, 0.029)
Ferritin, nmol/L	-0.004 (-0.008, -0.001)*	-0.001 (-0.002, 0.001)	-0.001 (-0.003, 0.002)
TIBC, µmol/L	0.077 (-0.002, 0.155)	0.037 (-0.026, 0.099)	0.037 (-0.026, 0.101)
Transferrin saturation, %	-0.011 (-0.053, 0.032)	-0.012 (-0.055, 0.032)	-0.009 (-0.052, 0.035)

Model 1 was for crude values from an unadjusted model. Model 2 was adjusted for age, region, education level, parity, and trimester. Model 3 was adjusted for model 2 plus daily nutrient intake such as protein (g and % of energy), folate (µg), and iron (mg). * *p* ≤ 0.05. CI: confidence interval; DP: dietary pattern; TIBC: total iron-binding capacity.

**Table 5:** Generalized linear regression analysis for the association of vegetables and fruits DP (DP-3) with serum anemia-related biomarkers among pregnant women with pre-pregnancy overweightness or obesity (*n* = 436)

Variables	Model 1	Model 2	Model 3
	β (95% CI)	β (95% CI)	β (95% CI)
Folate, nmol/L	0.039 (0.021, 0.051)*	0.049 (0.022, 0.065)*	0.056 (0.034, 0.076)*
Vitamin B_12_, pmol/L	-0.055 (-0.564, 0.453)	0.066 (-0.421, 0.554)	0.129 (-0.365, 0.623)
25(OH) Vitamin D, nmol/L	0.012 (-0.064, 0.088)	-0.019 (-0.094, 0.057)	-0.034 (-0.110, 0.041)
Hemoglobin, mmol/L	-0.004 (-0.008, 0.001)	-0.002 (-0.006, 0.003)	-0.002 (-0.006, 0.003)
Iron, µmol/L	-0.031 (-0.052, -0.010)*	-0.019 (-0.040, 0.002)	-0.020 (-0.041, 0.001)
Ferritin, nmol/L	-0.016 (-0.028, -0.009)*	-0.015 (-0.026, -0.008)*	-0.016 (-0.025, -0.007)*
TIBC, µmol/L	0.062 (-0.001, 0.126)	0.042 (-0.009, 0.093)	0.045 (-0.006, 0.097)
Transferrin saturation, %	0.003 (-0.032, 0.038)	0.001 (-0.035, 0.036)	0.002 (-0.034, 0.038)

Model 1 was for crude values from an unadjusted model. Model 2 was adjusted for age, region, education level, parity, and trimester. Model 3 was adjusted for model 2 plus daily nutrient intake such as protein (g and % of energy), folate (µg), and iron (mg). * *p* ≤ 0.05. CI: confidence interval; DP: dietary pattern; TIBC: total iron-binding capacity.

**Table 6:** Generalized linear regression analysis for the association of animal DP (DP-4) with serum anemia-related biomarkers among pregnant women with pre-pregnancy overweightness or obesity (*n* = 436)

Variables	Model 1	Model 2	Model 3
	β (95% CI)	β (95% CI)	β (95% CI)
Folate, nmol/L	0.002 (-0.071, 0.072)	0.025 (-0.043, 0.092)	0.024 (-0.046, 0.093)
Vitamin B_12_, pmol/L	0.244 (0.066, 0.509)*	0.248 (0.073, 0.567)*	0.321 (0.089, 0.571)*
25(OH) Vitamin D, nmol/L	0.053 (-0.037, 0.142)	0.034 (-0.054, 0.122)	0.009 (-0.080, 0.098)
Hemoglobin, mmol/L	0.017 (-0.008, 0.023)	0.018 (-0.006, 0.024)	0.020 (-0.007, 0.040)
Iron, µmol/L	0.015 (0.007, 0.026)*	0.016 (0.009, 0.040)*	0.025 (0.017, 0.049)*
Ferritin, nmol/L	0.014 (0.008, 0.028)*	0.016 (0.009, 0.032)*	0.022 (0.009, 0.046)*
TIBC, µmol/L	-0.068 (-0.088, 0.143)	-0.033 (-0.057, 0.093)	-0.038 (-0.063, 0.100)
Transferrin saturation, %	0.002 (-0.039, 0.043)	0.002 (-0.039, 0.043)	0.005 (-0.037, 0.047)

Model 1 was for crude values from an unadjusted model. Model 2 was adjusted for age, region, education level, parity, and trimester. Model 3 was adjusted for model 2 plus daily nutrient intake such as protein (g and % of energy), folate (µg), and iron (mg). * *p* ≤ 0.05. CI: confidence interval; DP: dietary pattern; TIBC: total iron-binding capacity.

**Table 7:** Odds ratios for low serum anemia-related biomarkers across the tertiles of mushrooms, roots, and dairy DP (DP-1) by binomial logistic regression analysis among pregnant women with pre-pregnancy overweightness or obesity (*n* = 436)

	Mushrooms, roots, and dairy DP (DP-1)							
Variables	Model 1 OR (95% CI)			Model 2 OR (95% CI)			Model 3 OR (95% CI)	
	T2	T3		T2	T3		T2	T3
Folate, nmol/L	0.508 (0.296, 0.872)*	0.482 (0.280, 0.830)*		0.637 (0.349, 1.163)	0.395 (0.214, 0.730)*		0.650 (0.354, 1.192)	0.378 (0.202, 0.709)*
Vitamin B_12_, pmol/L	0.952 (0.469, 1.930)	0.944 (0.466, 1.915)		0.972 (0.455, 2.077)	0.853 (0.407, 1.788)		1.013 (0.472, 2.178)	0.895 (0.418, 1.194)
25(OH) Vitamin D, nmol/L	0.856 (0.513, 1.438)	0.693 (0.419, 1.144)		0.860 (0.503, 1.470)	0.757 (0.451, 1.272)		0.813 (0.492, 1.178)	0.705 (0.418, 1.114)
Hemoglobin, mmol/L	0.602 (0.316, 1.145)	1.291 (0.735, 2.267)		0.806 (0.402, 1.616)	1.214 (0.661, 2.231)		0.821 (0.408, 1.654)	1.257 (0.676, 2.338)
Iron, µmol/L	0.819 (0.588, 1.187)	0.836 (0.527, 1.324)		0.660 (0.406, 1.073)	0.775 (0.480, 1.251)		0.663 (0.406, 1.083)	0.757 (0.463, 1.238)
Ferritin, nmol/L	0.806 (0.604, 0.976)	1.413 (0.891, 2.243)		1.188 (0.693, 2.036)	1.295 (0.756, 2.221)		1.174 (0.683, 2.016)	1.245 (0.721, 2.149)
TIBC, µmol/L	0.005 (0.004, 0.006)	0.005 (0.004, 0.006)		0.004 (0.003, 0.005)	0.004 (0.003, 0.005)		0.004 (0.02, 0.005)	0.004 (0.02, 0.005)
Transferrin saturation, %	1.030 (0.649, 1.633)	0.934 (0.590, 1.479)		1.013 (0.631, 1.625)	0.932 (0.583, 1.489)		1.009 (0.628, 1.621)	0.887 (0.551, 1.428)

Model 1 was for crude values from an unadjusted model. Model 2 was adjusted for age, region, education level, parity, and trimester. Model 3 was adjusted for model 2 plus daily nutrient intake such as protein (g and % of energy), folate (µg), and iron (mg). Serum anemia-related variables were categorized into two levels based on serum cutoff values: folate, 13.6 nmol/L (6 ng/mL); vitamin B_12_, 149.8 pmol/L (203 pg/mL); 25(OH) vitamin D, 75 nmol/L (30 ng/mL); hemoglobin, 6.52 mmol/L (10.5 g/dL); iron, 10.7 µmol/L (60 µg/dL), ferritin, 0.034 nmol/L (15 ng/mL); TIBC, 42.96 µmol/L (240 µg/dL); and transferrin saturation, 16%. Dietary pattern scores were classified into tertiles: T1 (reference), 0.49-13.68; T2, > 13.90-21.09; and T3 > 21.12-136.20. * *p* ≤ 0.05. CI: confidence interval; DP: dietary pattern; ORs: Odds ratios; TIBC: total iron-binding capacity.

**Table 8:** Odds ratios for low serum anemia-related biomarkers across the tertiles of vegetables and fruits DP (DP-3) by binomial logistic regression analysis among pregnant women with pre-pregnancy overweightness or obesity (*n* = 436)

	Vegetables and fruits DP (DP-3)							
Variables	Model 1 OR (95% CI)			Model 2 OR (95% CI)			Model 3 OR (95% CI)	
	T2	T3		T2	T3		T2	T3
Folate, nmol/L	0.610 (0.510, 0.861)*	0.552 (0.469, 0.8.97)*		0.852 (0.524, 0.942)*	0.763 (0.450, 0.895)*		0.756 (0.430, 0.915)*	0.462 (0.342, 0.714)*
Vitamin B_12_, pmol/L	1.077 (0.488, 2.379)	2.115 (1.036, 4.321)*		1.036 (0.458, 2.342)	1.901 (0.902, 4.004)		1.062 (0.466, 2.422)	1.975 (0.922, 4.231)
25(OH) Vitamin D, nmol/L	0.835 (0.509, 1.369)	1.107 (0.665, 1.842)		0.898 (0.537, 1.500)	1.303 (0.765, 2.219)		0.953 (0.565, 1.606)	1.515 (0.873, 2.630)
Hemoglobin, mmol/L	0.852 (0.456, 1.592)	1.470 (0.825, 2.619)		0.754 (0.386, 1.473)	1.100 (0.588, 2.056)		0.728 (0.370, 1.434)	1.081 (0.572, 2.043)
Iron, µmol/L	1.348 (0.845, 2.149)	1.703 (1.068, 2.716)*		1.319 (0.815, 2.133)	1.456 (0.897, 2.363)*		1.315 (0.808, 2.139)	1.435 (0.876, 2.352)
Ferritin, nmol/L	1.377 (0.867, 2.187)	2.390 (1.491, 3.833)*		1.460 (0.857, 2.488)	2.414 (1.391, 4.189)*		1.458 (0.853, 2.493)	2.367 (1.356, 4.132)*
TIBC, µmol/L	0.05 (0.03, 0.07)	0.05 (0.03, 0.07)		0.003 (0.002, 0.004)	0.003 (0.002, 0.004)		0.003 (0.001, 0.004)	0.003 (0.001, 0.004)
Transferrin saturation, %	0.705 (0.443, 1.121)	0.623 (0.391, 0.991)*		0.720 (0.450, 1.152)	0.632 (0.392, 1.017)		0.713 (0.445, 1.143)	0.601 (0.371, 0.975)

Model 1 was for crude values from an unadjusted model. Model 2 was adjusted for age, region, education level, parity, and trimester. Model 3 was adjusted for model 2 plus daily nutrient intake such as protein (g and % of energy), folate (µg), and iron (mg). Serum anemia-related variables were categorized into two levels based on serum cutoff values: folate, 13.6 nmol/L (6 ng/mL); vitamin B_12_, 149.8 pmol/L (203 pg/mL); 25(OH) vitamin D, 75 nmol/L (30 ng/mL); hemoglobin, 6.52 mmol/L (10.5 g/dL); iron, 10.7 µmol/L (60 µg/dL), ferritin, 0.034 nmol/L (15 ng/mL); TIBC, 42.96 µmol/L (240 µg/dL); and transferrin saturation, 16%. Dietary pattern scores were classified into tertiles: T1 (reference), 5.37-49.06; T2, > 49.50-77.47; and T3 > 77.54-121.80. * *p* ≤ 0.05. CI: confidence interval; DP: dietary pattern; ORs: Odds ratios; TIBC: total iron-binding capacity.

**Table 9:** Odds ratios for low serum anemia-related biomarkers across the tertiles of animal DP (DP-4) by binomial logistic regression analysis among pregnant women with pre-pregnancy overweightness or obesity (*n* = 436)

	Animal DP (DP-4)						
Variables	Model 1 OR (95% CI)		Model 2 OR (95% CI)		Model 3 OR (95% CI)		
	T2	T3	T2	T3	T2	T3	
Folate, nmol/L	0.617 (0.354, 1.075)	0.931 (0.552, 1.572)	0.598 (0.325, 1.099)	0.702 (0.392, 1.259)	0.600 (0.324, 1.111)	0.698 (0.386, 1.262)	
Vitamin B_12_, pmol/L	0.827 (0.449, 1.213)	0.840 (0.564, 1.284)	0.626 (0.413, 0.852)*	0.615 (0.470, 0.819)*	0.705 (0.511, 0.874)*	0.456 (0.302, 0.629)*	
25(OH) Vitamin D, nmol/L	1.400 (0.847, 2.315)	1.172 (0.715, 1.920)	1.394 (0.828, 2.348)	1.235 (0.740, 2.060)	1.449 (0.851, 2.469)	1.436 (0.845, 2.439)	
Hemoglobin, mmol/L	0.806 (0.429, 1.217)	1.027 (0.860, 1.214)	0.785 (0.400, 1.138)	0.953 (0.675, 1.326)	0.775 (0.403, 1.167)	0.877 (0.684, 2.384)	
Iron, µmol/L	0.802 (0.639, 0.959)*	0.804 (0.642, 0.962)*	0.805 (0.640, 0.964)*	0.809 (0.654, 0.968)*	0.806 (0.651, 0.965)*	0.810 (0.673, 0.967)*	
Ferritin, nmol/L	0.832 (0.715, 1.092)	0.837 (0.754, 1.153)	0.863 (0.698, 1.140)	0.902 (0.750, 1.146)	0.865 (0.779, 1.167)	0.927 (0.756, 1.238)	
TIBC, µmol/L	0.005 (0.003, 0.007)	0.005 (0.003, 0.007)	0.005 (0.002, 0.006)	0.004 (0.002, 0.005)	0.004 (0.002, 0.005)	0.003 (0.002, 0.004)	
Transferrin saturation, %	0.882 (0.556, 1.399)	0.758 (0.478, 1.203)	0.879 (0.551, 1.405)	0.759 (0.475, 1.211)	0.872 (0.545, 1.397)	0.730 (0.454, 1.172)	
							

Model 1 was for crude values from an unadjusted model. Model 2 was adjusted for age, region, education level, parity, and trimester. Model 3 was adjusted for model 2 plus daily nutrient intake such as protein (g and % of energy), folate (µg), and iron (mg). Serum anemia-related variables were categorized into two levels based on serum cutoff values: folate, 13.6 nmol/L (6 ng/mL); vitamin B_12_, 149.8 pmol/L (203 pg/mL); 25(OH) vitamin D, 75 nmol/L (30 ng/mL); hemoglobin, 6.52 mmol/L (10.5 g/dL); iron, 10.7 µmol/L (60 µg/dL), ferritin, 0.034 nmol/L (15 ng/mL); TIBC, 42.96 µmol/L (240 µg/dL); and transferrin saturation, 16%. Dietary pattern scores were classified into tertiles: T1 (reference), 3.56-28.29; T2, > 28.41-46.67; and T3 > 46.74-162.00. * *p* ≤ 0.05. CI: confidence interval; DP: dietary pattern; ORs: Odds ratios; TIBC: total iron-binding capacity.

## Abbreviations

BMI: body mass index
CI: confidence interval
DP: dietary pattern
FFQ: food frequency questionnaire
Hb: hemoglobin
OR: odds ratio
PCA: principal component analysis
RBC: red blood cells
TIBC: total iron-binding capacity
