# Supplementary material for: Association of nutrient intake and dietary patterns with serum folate and anemia-related biomarkers in Taiwanese pregnant women with pre-pregnancy overweightness or obesity
Source: Int J Med Sci. 2025 Feb 28;22(7):1630–9. doi: 10.7150/ijms.108760 (PMC11905261; doi:10.7150/ijms.108760)
Supplement: Supplementary file 1 — Supplementary tables. [file ijmsv22p1630s1.pdf]

Supplementary Table 1. Food groups and subgroups for dietary evaluation

Food groups		Subgroups
1. Dry rice, porridge and noodle products	1.	Rice and grains
	2.	Rice porridge
	3.	Noodles and related products
	4.	Rice milk
2. Breakfast cereals, oats, buns, glutinous rice, and related products	5.	Breakfast cereals, oats, and related products
	6.	Sweet buns and red beans
	7.	Salty steamed buns
	8.	Glutinous rice
	9.	Glutinous rice desserts
3. Bread and its products	10.	Bread and its products
4. Chinese dim sun and dumplings	11.	Chinese savory cake
	12.	Chinese buns and rolls
	13.	Dumplings
5. Roots, carrots, and tubers	14.	Root and tubers
	15.	Carrots
	16.	Root vegetables
	17.	Bamboo shoot
	18.	Melon
6. Eggs	19.	Eggs
7. Milk and milk products	20.	Fresh milk, milk powder

	21. Flavored milk
	22. Yogurt
	23. Cheese
8. Light-colored vegetables	24. Light-colored vegetables
9. Dark-colored vegetables	25. Dark-colored vegetables
10. Mushrooms and related products	26. Mushrooms
11. Legumes and beans	27. Mung beans, black eye beans, speckled kidney beans, and other seed beans and their products
12. Sea plant food	28. Sea vegetables
	29. Kelp
13. Processed plant food	30. Pickled vegetables
14. Herbs and spices	31. Garlic/garlic paste, onion, ginger, chili, green garlic, cilantro, toon, and nine-story tower
15. Soybean and soybean products	32. Soy milk, honey/soy milk, tofu pudding, and other soy milk drinks or products
	33. Gluten and gluten sausages
	34. General soy products
	35. Soy-fermented foods
16. Nuts and seeds	36. Nuts and seeds
17. Fresh fruits	37. Fresh fruits
18. Pure fruit juice or fruit/vegetable	38. Pure fruit juice or fruit and vegetable juice

juice	39. Commercially available sugary vegetable juice
19. Canned fruits, dried fruits, and jam	40. Canned fruits
	41. Dehydrated fruits, pickled fruits, and candied fruits
	42. Jam
20. Fish, shellfish, and seafood	43. Fish (including all fish)
	44. Aquatic products eaten with bones or shell
	45. Edible sea snails and mollusks
	46. Other seafood except fish and mollusks
	47. Deep water fish
21. Processed seafood products	48. Roe of marine animals
	49. Processed seafood products
22. Poultry meat	50. Poultry meat
23. Livestock meat	51. Lean livestock meat
	52. Livestock semi-fat meat
24. Liver, organs, and blood products	53. Liver
	54. Other internal organs besides the liver
	55. Poultry and livestock blood
	56. Other parts of poultry and livestock
25. Processed meat products	57. Processed meat products
	58. Processed meat paste products

26. Salt	59. Salt

Supplementary Table 2. Spearman's correlation coefficients between supplement intake and serum folate levels among pregnant women with pre-pregnancy overweight or obesity (*n* = 436)

Supplement intake, *n* (%)	Serum folate	
	ρ	*p*
Milk powder, 65 (14.8%)	–	–
Multivitamins/multiminerals, 259 (59.1%)	0.39	< 0.001
Folate (µg), 197 (45.0%)	0.50	0.042
Vitamin B complex, 77 (17.6%)	–	–
Vitamin D, 47 (10.7%)	–	–
Iron, 48 (11.0%)	–	–
Calcium (µg), 193 (44.1%)	0.12	0.043

The symbol of – shows no data evaluated because the consumption frequency was below 20%.

Supplementary Table 3. Generalized linear regression analysis for the association of processed and plant-based DP (DP-2) with serum anemia-related biomarkers among pregnant women with pre-pregnancy overweight or obesity (*n* = 436)

Variables	Model 1	Model 2	Model 3
	β (95% CI)	β (95% CI)	β (95% CI)
Folate, nmol/L	0.004 (-0.001, 0.009)	0.072 (-0.040, 0.183)	0.072 (-0.041, 0.185)
Vitamin B_{12}, pmol/L	-0.279 (-1.268, 0.709)	-0.505 (-1.443, 0.433)	-0.415 (-1.367, 0.537)
25(OH) Vitamin D, nmol/L	0.081 (-0.066, 0.229)	0.054 (-0.091, 0.198)	0.026 (-0.120, 0.173)
Hemoglobin, mmol/L	-0.004 (-0.012, 0.005)	-0.005 (-0.013, 0.004)	-0.005 (-0.014, 0.004)
Iron, µmol/L	-0.008 (-0.049, 0.034)	-0.012 (-0.052, 0.029)	-0.009 (-0.050, 0.032)
Ferritin, nmol/L	-0.001 (-0.002, 0.001)	-0.001 (-0.002, 0.002)	-0.001 (-0.001, 0.002)
TIBC, µmol/L	0.013 (-0.137, 0.111)	0.013 (-0.086, 0.112)	0.013 (-0.087, 0.114)
Transferrin saturation, %	-0.030 (-0.097, 0.037)	-0.024 (-0.092, 0.045)	-0.019 (-0.088, 0.050)

Model 1 was for crude values from an unadjusted model. Model 2 was adjusted for age, region, education level, parity, and trimester. Model 3 was adjusted for model 2 plus daily nutrient intake such as protein (g and % of energy), folate (µg), and iron (mg). CI: confidence interval; DP: dietary pattern; ORs: Odds ratios; TIBC: total iron-binding capacity.

Supplementary Table 4. Odds ratios for low serum anemia-related biomarkers across the tertiles of processed and plant-based DP (DP-2) by binomial logistic regression analysis among pregnant women with pre-pregnancy overweight or obesity (*n* =436)

	Processed and plant-based DP (DP-2)					
Variables	Model 1 OR (95% CI)		Model 2 OR (95% CI)		Model 3 OR (95% CI)	
	T2	T3	T2	T3	T2	T3
Folate, nmol/L	0.534 (0.314, 0.908)*	0.451 (0.260, 0.781)*	0.566 (0.315, 1.015)	0.475 (0.256, 0.880)*	0.552 (0.303, 1.005)	0.412 (0.214, 0.791)*
Vitamin B_{12}, pmol/L	0.573 (0.275, 1.193)	0.830 (0.422, 1.633)	0.555 (0.259, 1.188)	0.899 (0.439, 1.841)	0.559 (0.257, 1.218)	0.890 (0. 419, 1.892)
25(OH) Vitamin D, nmol/L	0.890 (0.533, 1.484)	0.706 (0.427, 1.166)	0.919 (0.542, 1.558)	0.727 (0.431, 1.226)	1.037 (0.602, 1.787)	0.881 (0.508, 1.529)
Hemoglobin, mmol/L	0.900 (0.502, 1.613)	0.826 (0.457, 1.495)	1.037 (0.552, 1.948)	0.996 (0.521, 1.904)	1.061 (0.559, 2.016)	0.977 (0.498, 2.043)
Iron, µmol/L	1.091 (0.686, 1.735)	1.201 (0.755, 1.901)	1.231 (0.761, 1.991)	1.258 (0.776, 2.039)	1.288 (0.786, 2.111)	1.229 (0.742, 2.036)
Ferritin, nmol/L	0.893 (0563, 1.417)	0.789 (0.497, 1.253)	0.842 (0.495, 1.432)	0.861 (0.498, 1.488)	0.777 (0.450, 1.340)	0.793 (0.448, 1.406)
TIBC, µmol/L	0.500 (0.200, 1.300)	0.450 (0.201, 1.135)	0.256 (0.078 1.384)	0.955 (0.428, 1.642)	0.960 (0.430, 1.645)	1.291 (0.596, 1.894)
Transferrin saturation, %	1.473 (0.927, 2.340)	1.165 (0.734, 1.848)	1.481 (0.926, 2.371)	1.140 (0.712, 1.825)	1.426 (0.883, 2.301)	1.074 (0.659, 1.752)

Model 1 was for crude values from an unadjusted model. Model 2 was adjusted for age, region, education level, parity, and trimester. Model 3 was adjusted for model 2 plus daily nutrient intake such as protein (g and % of energy), folate (µg), and iron (mg). Serum anemia-related variables were categorized into two levels based on serum cutoff values: folate, 13.6 nmol/L (6 ng/mL); vitamin B_{12}, 149.8 pmol/L (203 pg/mL); 25(OH) vitamin D, 75 nmol/L (30 ng/mL); hemoglobin, 6.52 mmol/L (10.5 g/dL); iron, 10.7 µmol/L (60 µg/dL), ferritin, 0.034 nmol/L (15 ng/mL); TIBC, 42.96 µmol/L (240 µg/dL); and transferrin saturation, 16%. Dietary pattern scores were classified into tertiles: T1 (reference), 1.26–10.64; T2, > 10.65–19.48; and T3 > 19.05–158.77. * $p \leq 0.05$. CI: confidence interval; DP: dietary pattern; ORs: Odds ratios; TIBC: total iron-binding capacity.
